# Prediction of lymph node status in completely resected IIIa/N2 small cell lung cancer: importance of subcarinal station metastases

**DOI:** 10.1186/s13019-019-0886-y

**Published:** 2019-03-29

**Authors:** Rong Qiao, Runbo Zhong, Jianlin Xu, Yanwei Zhang, Bo Zhang, Shuyuan Wang, Yuqing Lou, Dongfang Chen, Qing Chang, Yizhuo Zhao, Baohui Han

**Affiliations:** Department of Pulmonary Medicine, Shanghai Chest Hospital, Shanghai Jiao Tong University, West Huaihai Road 241#, Shanghai, 200030 People’s Republic of China

**Keywords:** Small cell lung cancer, N2 disease, Subcarinal lymph node, Prognostic factors

## Abstract

**Background:**

The aim of this study was to determine the prognostic value of lymph node status in patients with pathologic N2 (pN2) stage IIIA small cell lung cancer (SCLC).

**Methods:**

A total of 163 consecutive pN2 stage IIIA SCLC patients who underwent pulmonary resections and systematic lymphadenectomies at Shanghai Chest Hospital between January 2006 and June 2014 were enrolled. We retrospectively analyzed the potential clinicopathologic factors that influenced survival, including the node levels (single or multiple-station) and the node-spreading patterns (skip N2 or non-skip N2). The prognostic significance was examined by Cox regression analysis.

**Results:**

The median overall survival (OS) was 23.7 months. Multiple-station lymph node metastasis indicated a poorer prognosis than single-station involvement (*p* = 0.003). Skip metastasis did not appear to influence survival (*p* = 0.099). With respect to the station of lymph node metastasis, the OS was only related to the involvement of the subcarinal node, regardless of tumor location (*p* < 0.05). Multivariate analysis showed two statistically significant risk factors for survival, including multiple-station lymph node and subcarinal node metastasis (hazard ratio [HR] = 1.76, 95% confidence interval [CI]:1.11–2.78, *p* = 0.015; HR = 1.61, 95% CI: 1.03–2.50, *p* = 0.036, respectively).

**Conclusions:**

Multiple-station N2 metastasis and involvement of the subcarinal node predicted poor prognosis in pN2 stage IIIA SCLC patients, which may profoundly influence therapeutic decisions.

## Introduction

Lung cancer is the most frequently diagnosed cancer and the leading cause of cancer-related deaths worldwide [1]. Small cell lung cancer (SCLC) accounts for ~ 15% of all newly diagnosed lung cancer cases [2]. SCLC is more aggressive than non-small cell lung cancer (NSCLC) because of the more rapid doubling time, higher growth fraction, and earlier metastatic spread [3].

Chemoradiation therapy is recommended as standard management in patients with limited-stage SCLC. However, several large population database series have recently reported favorable survival outcomes in limited-stage patients who underwent surgery combined with non-surgical treatment, even for stage IIIA disease [4, 5]. Thus, the diagnosis of SCLC is sometimes difficult from small specimens obtained by bronchoscopy and/or needle biopsy. Most patients did not have a confirmed diagnosis of SCLC pre- or intra-operatively, until a surgically-resected specimen showed SCLC, even though there was no suspicion of N2 lymph node (LN) metastasis. Indeed, surgical resection is increasingly used for pN2 IIIA SCLC; however, the prognostic impact of LN involvement in surgically-treated SCLC has been rarely evaluated to date. Therefore, to achieve better local control, ascertaining prognostic factors has been especially important to guide post-operative multidisciplinary treatment and helpful to identify appropriate sub-groups of N2 patients who can benefit from surgical intervention.

In this study we investigated the node levels and spreading patterns in patients with completely resected SCLC with pathologic N2 (pN2) stage IIIA, and to identify the subgroups which may affect post-operative survival.

## Materials and methods

### Patients

We retrospectively reviewed the patients with pN2 stage IIIA SCLC who underwent surgical resection at Shanghai Chest Hospital between January 2006 and June 2014. Patients with post-operative follow-up for at least 3 months, Eastern Cooperative Oncology Group performance status 0–1, a single primary tumor, systematic mediastinal nodal dissection, negative resection margins, and no pre-operative neoadjuvant therapy (chemotherapy and/or radiation therapy) were included. The current study was conducted with approval of the Institutional Review Board of Shanghai Chest Hospital.

We recorded the following clinicopathologic variables in the analysis: age; gender; smoking history; positron emission tomography (PET) scan; tumor endoscopy (central and peripheral); tumor location (upper, middle, and lower lobes); pre-operative diagnosis; type of resection; histologic type; visceral pleura invasion; lymphovascular invasion (LVI); pathologic tumor size (cm); post-surgical N stage; number and pattern of LN involvement; and administration of induction and/or adjuvant treatments.

### Classification and definition of pathologic N status

Surgical-pathologic staging was assigned according to the 7th edition of the tumor–node–metastasis classification system. N2 LNs were classified according to the LN map published by the 2009 International Association for the Study of Lung Cancer [6]. Metastasis to the pN2 was classified as follows:

(1) according to the node levels (single-station [metastasis to one N2 station] or multiple-station [metastases to 2 or more N2 stations] N2 metastases); and (2) according to the node-spreading patterns (skip [N2 lymph node metastases without any N1 node involvement] or non-skip [N2 lymph node metastases with N1 node involvement] N2 metastases).

### Statistical analysis

Overall survival (OS) was defined as the time from surgery to death from any cause or the last follow-up date. Statistical analysis was performed using a χ2 test for categorical variables and an unpaired t-test for continuous variables. Survival curves was estimated using the Kaplan–Meier method and compared by a log-rank test. Univariate analysis used the following outcome variables: patient age, sex, smoking status, PET (positron emission tomography) scan, histology, tumor endoscopy, visceral pleura invasion, lymphovascular invasion, tumor size, node levels, node-spreading patterns, subcarinal LN metastasis, cycles of chemotherapy, PORT to the lung and PCI. Multivariate survival analysis using the Cox proportional hazards regression model was performed to assess the prognostic significance of pN2 sub-classification, including clinicopathologic variables. Binary logistic regression was used to analyse independent risk factors correlated to subcarinal lymph node metastasis. A two-sided *p*-value < 0.05 was defined as statistically significant. Statistical analyses were performed using SPSS software (version 22; SPSS, Inc., Chicago, IL, USA).

## Results

### Patient characteristics

**Fig. 1:**
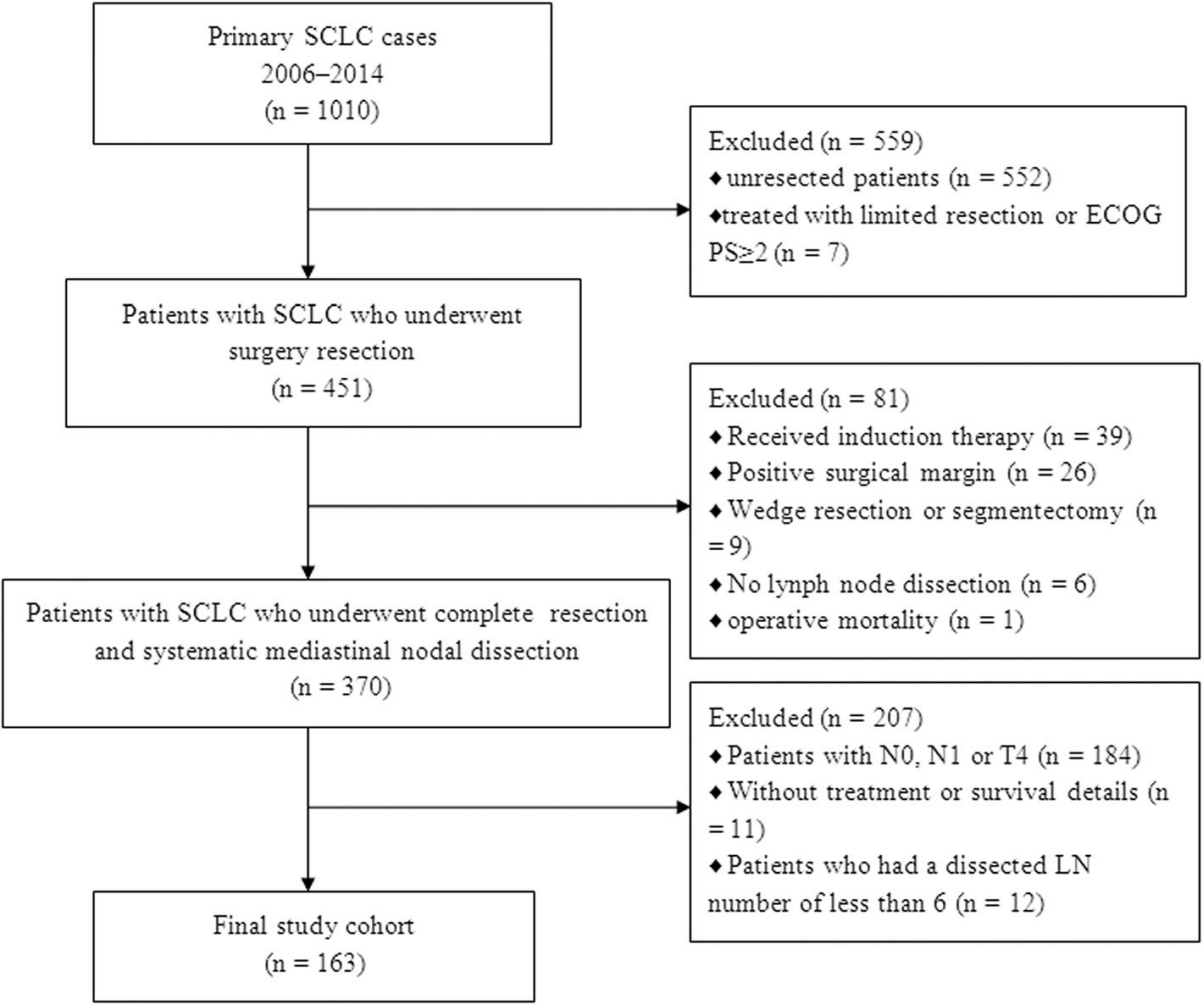
Flow chart of patient selection. Abbreviations: SCLC, small cell lung cancer; ECOG PS, Eastern Cooperative Oncology Group performance status

A total of 163 consecutive patients with pN2 stage IIIA SCLC were included in this retrospective analysis (Fig. 1). The demographic data of all patients are summarized in Table 1. Among the 163 patients, 132 (81.0%) were male and 31(19.0%) were female. The mean age was 58.7 ± 8.7 years, with an age range from 33 to 79 years. All of the patients had a bronchoscopy examination with bronchial brushing. 63 (38.7%) patients receive PET-staged to assess the mediastinal lymph nodes. 102 (62.5%) patients underwent preoperative biopsy by CT-guided transthoracic needle aspiration and/or bronchoscopy. 40 (24.5%) patients were preoperatively diagnosed with SCLC, 37 (22.7%) patients were diagnosed with other types of cancer (included unclassified carcinoma), and 86 (52.8%) patients received CT-guided transthoracic needle aspiration and/or bronchoscopy (including EBUS) with negative results. The pathologic sub-type was pure SCLC in 117 patients (71.8%) and combined SCLC in the remaining 46 patients. The most frequent procedure was a lobectomy (81.6%). One hundred eleven patients (68.1%) received post-operative radiotherapy (PORT) to the lung. The median delivered dose was 52 Gy (range, 30–80 Gy). Fifty-five patients (33.7%) received prophylactic cranial irradiation (PCI). All patients received adjuvant chemotherapy within 28–45 days after surgery. Different chemotherapy regimens were used: 96 patients received cisplatin plus etoposide, 60 patients received etoposide plus carboplatin, and only 7 patients received etoposide alone. 67 patients (41.1%) received more than four cycles of adjuvant chemotherapy. Single- and multiple-station N2 was present in 85 (52.1%) and 78 patients (47.9%), respectively. Skip metastases were recorded in 26 patients (16.0%).

**Table 1:** Baseline clinicopathologic characteristics of 163 patients

Variables	All patients	Subcarinal node (−)	Subcarinal node (+)	*p* value
	*n* = 163 (%)	*n* = 89	*n* = 74	
Age at surgery				
< 60 years	89 (54.6%)	49	40	0.898
≥ 60 years	74 (45.4%)	40	34	
Gender				
Male	132 (81.0%)	74	58	0.440
Female	31 (19.0%)	15	16	
Smoking status				
Smoker	121 (74.2%)	67	54	0.737
Never-smoker	42 (25.8%)	22	20	
PET scan				
Yes	63 (38.7%)	34	29	0.897
No	100 (61.3%)	55	45	
Preopeative biopsy				
SCLC	40 (24.5%)	20	20	0.793
Other types of cancer	37 (22.7%)	20	17	
Cancer not diagnosed	86 (52.8%)	49	37	
Surgery type				
Lobectomy	133 (81.6%)	72	61	0.801
Pneumonectomy	30 (18.4%)	17	13	
Histology				
Pure SCLC	117 (71.8%)	67	50	0.276
Combined SCLC	46 (28.2%)	22	24	
Tumor location				
Upper lobe	84 (51.5%)	66	18	< 0.05
Middle lobe	12 (7.4%)	2	10	
Lower lobe	67 (41.1%)	21	46	
Tumor endoscopy				
Peripheral	66 (40.5%)	37	29	0.758
Central	97 (59.5%)	52	45	
Visceral pleura invasion				
Yes	23 (14.1%)	12	11	0.801
No	140 (85.9%)	77	63	
LVI				
Yes	44 (27.0%)	23	21	0.717
No	119 (73.0%)	66	53	
Tumor size (cm)	/	3.69 ± 1.36	4.54 ± 1.81	0.001
≤ 3	51 (31.3%)	34	17	0.005
> 3, ≤ 5	77 (47.2%)	44	33	
> 5	35 (21.5%)	11	24	
Cycles of Chemotherapy				
≤ 4	96 (58.9%)	50	46	0.440
> 4	67 (41.1%)	39	28	
PCI				
Yes	55 (33.7%)	33	22	0.323
No	108 (66.3%)	56	52	
PORT to the lung				
Yes	111 (68.1%)	65	46	0.138
No	52 (31.9%)	24	28	
Node levels				
Single-station N2	85 (52.1%)	59	26	< 0.05
Multiple-station N2	78 (47.9%)	30	48	
Node-spreading patterns				
Nonskip N2	137 (84.0%)	71	66	0.102
Skip N2	26 (16.0%)	18	8	

Abbreviations: *PET* positron emission tomography, *SCLC* small cell lung cancer, *LVI* lymphovascular invasion, *PORT* postoperative radiotherapy, *PCI*, prophylactic cranial irradiation, *HR* hazard ratio, *CI* confidence interval

### pN2 subclassification and survival

**Fig. 2:**
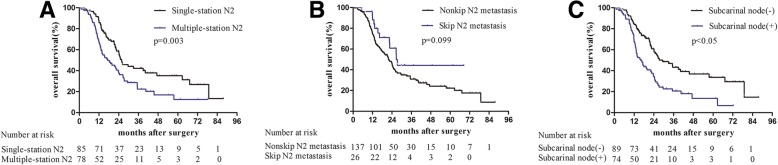
Kaplan-Meier analysis of the overall survival according to **a** node levels; **b** node-spreading patterns; **c** subcarinal node involvement

The overall follow-up ranged from 13 to 140 months with a median of 50 months. The median OS was 23.7 months (hazard ratio [HR], 1.33, 95% confidence interval [CI], 21.1–26.3) among all patients. The levels and locations of involved nodes affecting survival (Fig. 2). Although skip N2 metastasis was not associated with OS (*p* = 0.099), the node levels were significantly related with OS (*p* = 0.003). The OS was 25.71 months (HR, 2.88, 95% CI, 20.07–31.35) and 17.31 months (HR, 3.72, 95% CI, 10.01–24.61) for patients with single- and multiple-station N2 metastasis, respectively. Furthermore, the OS for patients without subcarinal LN metastasis (29.36 months; HR, 4.73, 95% CI, 20.09–38.63) was significantly longer than patients with subcarinal LN metastasis (16.14 months; HR, 2.11, 95% CI, 12.01–20.27; *p* < 0.05). The remaining LNs, including metastases of stations 2,3,4,5,6,8, and 9, were of no statistical significance with respect to OS (data not shown).

Univariate Cox analyses showed that tumor endoscopy, tumor size, LVI, PORT to the lung, PCI, N2 levels, and subcarinal LN metastasis were predictive of survival (Table 2). Multivariate analyses confirmed that PORT to the lung (yes versus no; HR, 1.53, 95% CI, 1.01–2.32, *p* = 0.041), PCI (yes versus no; HR, 2.10; 95% CI, 1.31–3.36, *p* = 0.002), node levels (single versus multiple; HR, 1.84, 95% CI, 1.19–2.84, *p* = 0.006), and subcarinal LN metastasis (no versus yes; HR, 1.57, 95% CI, 1.02–2.41, *p* = 0.037) were independent prognostic factors (Table 3).

**Table 2:** Univariate analysis for overall survival

Variables	Univariate Analysis	
	HR(95% CI)	*p* value
Age		
< 60 years	1	0.703
≥ 60 years	1.07 (0.73–1.57)	
Gender		
Male	1	0.790
Female	1.06 (0.65–1.74)	
Smoking status		
Smoker	1	0.690
Never-smoker	0.91 (0.59–1.40)	
PET scan		
Yes	1	0.681
No	1.08 (0.73–1.59)	
Histology		
Pure SCLC	1	0.486
Combined SCLC	1.16 (0.75–1.79)	
Tumor endoscopy		
Peripheral	1	0.005
Central	1.83 (1.20–2.78)	
Visceral pleura invasion		
Yes	1	0.624
No	0.86 (0.49–1.52)	
LVI		
Yes	1	0.047
No	0.66 (0.44–0.99)	
Tumor size (cm)		
≤ 3	1	
> 3, ≤ 5	1.88 (1.14–3.08)	0.012
> 5	2.31 (1.32–4.05)	0.003
Cycles of Chemotherapy		
≤ 4	1	0.079
> 4	0.70 (0.47–1.04)	
PORT to the lung		
Yes	1	0.003
No	1.81 (1.22–2.68)	
PCI		
Yes	1	0.004
No	1.89 (1.23–2.89)	
Node levels		
Single-station N2	1	0.003
Multiple-station N2	1.78 (1.21–2.61)	
Node-spreading patterns		
Nonskip N2	1	0.103
Skip N2	1.64 (0.90–3.01)	
Subcarinal LN metastasis		
No	1	< 0.05
Yes	2.08 (1.42–3.07)	

Abbreviations: *PET* positron emission tomography, *SCLC* small cell lung cancer, *LVI* lymphovascular invasion, *PORT* postoperative radiotherapy, *PCI* prophylactic cranial irradiation, *HR* hazard ratio, *CI* confidence interval

**Table 3:** Multivariate analysis for overall survival

Variables	Multivariate Analysis	
	HR(95% CI)	*p* value
Age		
< 60 years	1	0.411
≥ 60 years	1.19 (0.78–1.81)	
Gender		
Male	1	0.420
Female	1.34 (0.65–2.73)	
Smoking status		
Smoker	1	0.520
Never-smoker	0.81 (0.44–1.51)	
Tumor endoscopy		
Peripheral	1	0.017
Central	1.79 (1.11–2.90)	
LVI		
Yes	1	0.420
No	0.83 (0.53–1.29)	
Tumor size (cm)		
≤ 3	1	
> 3, ≤ 5	1.46 (0.84–2.52)	0.174
> 5	1.65 (0.74–3.64)	0.216
PORT to the lung		
Yes	1	0.041
No	1.53 (1.01–2.32)	
PCI		
Yes	1	0.002
No	2.10 (1.31–3.36)	
Node levels		
Single-station N2	1	0.006
Multiple-station N2	1.84 (1.19–2.84)	
Subcarinal LN metastasis		
No	1	0.037
Yes	1.57 (1.02–2.41)	

Abbreviations: *SCLC* small cell lung cancer, *LVI* lymphovascular invasion, *PORT* postoperative radiotherapy, *PCI* prophylactic cranial irradiation, *HR* hazard ratio, *CI* confidence interval

### Subcarinal LN metastasis

Subcarinal LN metastasis was observed in 74 (45.4%) of the 163 patients with N2 disease. Among the 36 patients with N2 disease of the right upper lobe, 10 (27.8%) had subcarinal LN. Similarly, of the 48 patients with N2 disease in the left upper lobe, 8 (16.7%) had subcarinal LN involvement. Of the 52 patients with N2 disease of the right middle and lower lobes, 39 (75%) had subcarinal LN involvement. Among the 27 patients with N2 in the left lower lobe, 17 (63%) had subcarinal LN involvement. The most common locations for the primary tumor in cases with subcarinal LN metastases were in the left lower (63%) and right lower lobes (74.4%).

Among the variables potentially associated with subcarinal LN metastasis, tumor location, tumor size, and node levels differed significantly between patients with and without subcarinal LN metastasis (Table 1). As indicated by the multivariate logistic regression analysis, the tumor size was not statistically significant independent risk factors predicting subcarinal LN involvement; however, tumor location (middle/lower versus upper; *p* < 0.05) and node levels (multiple versus single; p < 0.05) were associated with a 13.839-, 8.320-, and 4.041-fold increased risk of subcarinal LN metastasis, respectively (Table 4).

**Table 4:** Multivariate logistic regression analysis of factors correlated to subcarinal lymph node metastasis

Variables	OR	95%CI	*p* value
Tumour location			
Upper lobe^a^			
Middle lobe	13.83	2.58–74.19	0.002
Lower lobe	8.32	3.72–18.60	< 0.05
Node levels			
Single-station N2^a^			
Multiple-station N2	4.04	1.85–8.81	< 0.05

Abbreviations: *OR* odds ratio, *CI* confidence interval

^a^Reference category

## Discussion

To date, the prognostic impact of the involved lymph nodes in surgically-resected SCLC has rarely been evaluated, and the identification of patients who might benefit from more aggressive post-operative therapy remains a challenge. Therefore, an assessment of the prognostic characteristics of LN metastasis in patients with SCLC is very useful in selecting appropriate patients for surgery and guide effective adjuvant therapy.

We reviewed 163 consecutive patients who underwent pulmonary resections for pN2 IIIA SCLC. Only 24.5% of patients were diagnosed SCLC before surgery. In our opinion, the accuracy of the pre-operative diagnosis is important to help establish the best treatment strategy, and may influence survival of patients with SCLC.

In this study, the percentage of multiple-station N2 was 24.5%, and survival analysis showed a greater number of N2 LNs was associated with worse OS (*p* = 0.003). We also found that the worse prognostic value of multiple-station N2 involvement was highly significant (*p* = 0.015). There have been several reports that have shown patients with involvement of multiple-station N2 have a worse prognosis than patients with single-station N2 involvement in completely resected pN2 NSCLC [7–11]. These reports were in agreement with our present study in patients with SCLC. Involvement of multiple-station N2 may imply increased tumor burden in the lymphatic flow and opportunity of systemic spread of tumor cells, which can lead to early recurrence of tumors [12].

Skip N2 metastasis is thought to be derived from subpleural lymphatics that drain directly to the mediastinum [7]. The incidence of skip N2 metastases is 20–40% of all N2 diseases in resected NSCLC [13], and our study (16.0%) was slightly lower than these previous reports. In resected pN2 NSCLC, several studies have suggested an increased survival for skip metastases [14–17]; however, other reports with contradictory findings also exist [7, 18, 19]. In SCLC, Leuzzi et al. [20] found N0 N2-patients showed a worse cancer-specific survival compared to patients with combined N1 N2-involvement (N0 N2 [8 months] versus N1 N2 [22 months]; *p* = 0.04). Our data showed no statistically significant difference in survival between patients with skip and non-skip N2 metastases. Further studies with larger cohorts are needed to define the prognostic role of the node-spreading patterns.

Moreover, the locations of the LNs involved may also have prognostic significance. Okada et al. [21] suggested that the subcarinal LN is an independent prognostic factor among pN2 NSCLC patients with an upper lobe tumor (*p* = 0.023). Patients with subcarinal node involvement from right or left upper lobe tumors (*n* = 8) have a significantly worse prognosis than patients with metastases to the upper mediastinal or aortic nodes only (*n* = 70), and the 5-year survival for these patients was 0 and 37%, respectively. These results are in agreement with the results reported by others [22–24], confirming a poor outcome in NSCLC with subcarinal LN involvement. Few studies have investigated the prognostic value of subcarinal LN metastasis in patients with SCLC. Miyamoto et al. [25] suggested that the prognosis was significantly poorer in SCLC patients with subcarinal LN involvement than those without subcarinal LN involvement and pN2 disease (*p* = 0.0319) by univariate analysis, which was consistent with the previous reports involving NSCLC.

In the current study, it was surprising that subcarinal LN had a high incidence of involvement (45.4% [74 of 163]) in pN2 IIIA SCLC. Such a high incidence reflects the virtual situation that has been commonly neglected. Thus, subcarinal LN metastasis was significantly more common in patients with lower lobe or middle lobe cancers compared with upper lobe cancers (70.9% versus 21.4%, *p* < 0.05). In addition, we concluded that tumor location and node levels were significant variables for identifying patients with subcarinal LN metastasis. Our study showed that subcarinal LN metastasis is a predictive factor for worse OS in patients with pN2 IIIA SCLC regardless of tumor location. The median OS for patients with subcarinal LN metastasis was significantly shorter than patients without subcarinal LN metastasis (16.14 months versus 29.36 months, p < 0.05), the survival of 16.14 months was nearly the same with that reported for patients receiving chemoradiation alone in the presence of stage III/N2 disease. The National Cancer Data Base (NCDB) analysis found that compared to chemotherapy-based non-surgical treatment, surgery was associated with longer survival for SCLC patients with stage IIIA (median OS 21.7 vs. 16.0 months, *p* < 0.0001) and node positivity(N2+ 20.1 vs. 14.6 months *p* = 0.007) [26]. Because the prognosis of small cell lung cancer patients with subcarinal nodal disease is poor, accurate staging is important to direct patients to the most effective treatments, chemoradiation but not surgery may be more potent for these patients. Although subcarinal nodal biopsy is not essential to determine resectability, subcarinal LN should be dissected or sampled routinely during operations for SCLC to avoid understaging.

We have no ready explanation for the poor prognosis of patients with subcarinal LN metastases. We speculate that the subcarinal node might be significant as a common path where the lymphatic channels from various organs in the thorax meet, either directly or by means of lymphoid relays [27]. Our own study confirmed the importance of the subcarinal LN and the poor prognostic implications. The underlying reason for these results may be that subcarinal LN metastasis indicates a wider range of mediastinal involvement and widespread micro-metastasis via the lymphatic network. Thus, SCLC with subcarinal metastases might be more advanced and have a higher biological potential for spread than SCLC without subcarinal metastases in patients with pN2 disease.

Our study had several limitations. Specifically, the study was retrospective from a single institution with a small number of patients over a long study period, and lack of cohort design, which might cause selection bias. Second, there was no central pathologic review, although histologic specimens were evaluated by pathologists experienced in evaluating lung tumors. Third, there may have been a lack of uniformity because different surgeons performed pulmonary resections over a long period of time. The LN dissection number was also not consistent, which may introduce another bias. Therefore, we excluded patients who had a dissected LN number of < 6. Fourth, pre-operative PET scan, mediastinoscopy, and endobronchial ultrasound were not routinely performed for pathologic staging of suspicious nodes. Before surgery, patients with mediastinal lymph nodes received EBUS. If the biopsy of mediastinal lymph nodes was negative, patients were selected for an operation. In the current study, only 63 (38.7%) patients receive PET to assess the mediastinal lymph nodes, which is a source of potential weakness because some patients with a poor prognosis may be enrolled in this study. Further evaluation is needed to evaluate the impact of the LN status on survival of patients with pN2 IIIA SCLC and confirm our results.

## Conclusions

Our study showed that N2 levels and subcarinal LN metastasis were significant indicators of worse OS in patients with completely resected pN2 IIIA SCLC. The differences in survival between these sub-groups of patients suggest that they should be considered for more aggressive adjuvant therapy and different postsurgical follow-up strategies. Our data demonstrated the validity of avoiding surgery after identifying the sites (subcarinal level) of potential nodal metastases of SCLC with N2. Larger prospective clinical trials evaluating the role of the adjuvant therapies should divide patients into separate groups based on these factors.

## Abbreviations

CI: Confidence interval
ECOG: Eastern Cooperative Oncology Group
HR: Hazard ratios
LN: Lymph node
LVI: Lymphovascular invasion
NSCLC: Non-small cell lung cancer
OS: Overall survival
PCI: Prophylactic cranial irradiation
PET: Positron emission tomography
pN2: Pathologic N2
PORT: Postoperative radiotherapy
SCLC: Small cell lung cancer
